# Healthcare-provider perceptions of barriers to oxygen therapy for paediatric patients in three government-funded eastern Ugandan hospitals; a qualitative study

**DOI:** 10.1186/s12913-019-4129-7

**Published:** 2019-05-24

**Authors:** Jonathan W. Dauncey, Peter Olupot-Olupot, Kathryn Maitland

**Affiliations:** 1 Wellcome Trust Centre for Clinical Tropical Medicine and Department of Paediatrics, Faculty of Medicine, Imperial College, London, W2 1PG UK; 2 Department of Paediatrics, Mbale Regional Referral Hospital, Pallisa Road, PO Box 291, Mbale, Uganda; 3 Mbale Clinical Research Institute (MCRI), Plot 29-33 Pallisa Rd, P.O. Box 1966, Mbale, Uganda; 4 KEMRI-Wellcome Trust Research Programme, Centre for Geographic Medicine Research-Coast, PO Box 230, Kilifi, Kenya

**Keywords:** Oxygen, Paediatrics, Uganda, Africa

## Abstract

**Background:**

This study aimed to assess on-the-ground barriers to the provision of oxygen therapy for paediatric patients in three government-funded Eastern Ugandan district general hospitals (DGHs).

**Methods:**

Site visits to DGHs during March 2017 involved semi-structured interviews with medical officers, clinical officers, paediatric nurses and non-clinical staff (*n* = 29). MAXQDA qualitative data software was used to assist with response analysis.

**Results:**

The healthcare professionals reported that erratic electricity supplies, few and/or malfunctioning oxygen cylinders and concentrators, limited or no access to pulse oximetry, inadequate staffing and lack of continued professional training were key barriers to the delivery of oxygen therapy. Local populations were reportedly fearful of oxygen therapy and reluctant to consent for oxygen therapy to be administered to their children.

**Conclusion:**

According to healthcare providers in three Eastern Ugandan DGHs, numerous barriers exist to oxygen therapy for paediatric patients. Healthcare professionals reported lack of facilities and training to effectively deliver oxygen therapy. Quality improvement work prioritising oxygen therapy in government-funded district general hospitals should focus on oxygen supply and delivery issues on a site-specific level and sensitizing communities to the potential benefits of oxygen.

## Background

Alongside general protection, prevention and treatment strategies, including feeding, vaccination and reducing household air pollution, for those who develop severe pneumonia oxygen therapy has been identified as a key area for development in the integrated Global Action Plan for Pneumonia and Diarrhoea (GAPPD) [1]. This represents a World Health Organisation (WHO) and United Nations Children’s Fund (UNICEF) collaboration which aims to ‘end preventable childhood deaths due to pneumonia and diarrhoea by 2025’. In sub-Saharan Africa, pneumonia is attributed to 17% of all deaths in children under five, the largest killer of children in this region [2]. Understanding oxygen supply and delivery in this region is therefore fundamental to healthcare improvement.

**Fig. 1:**
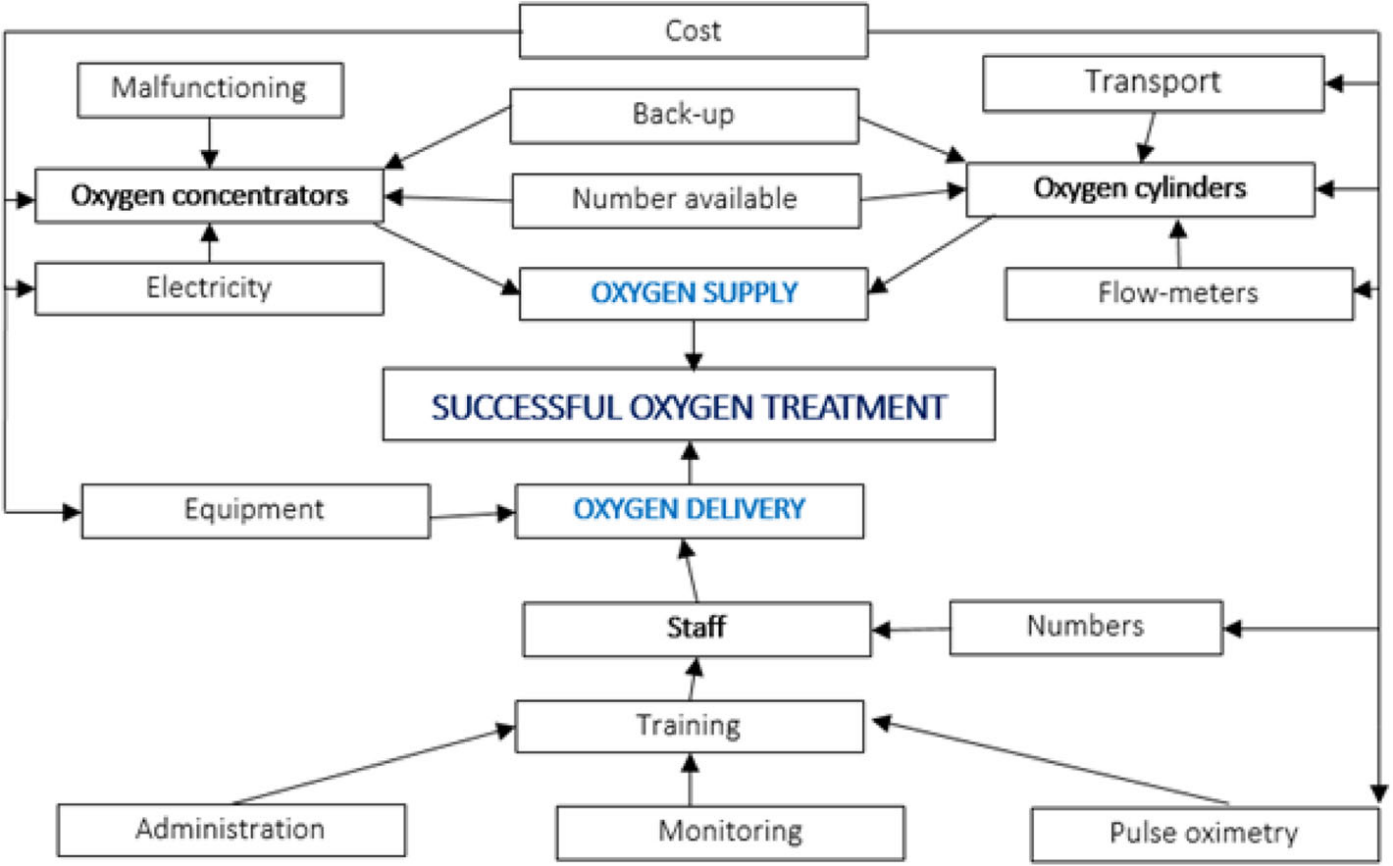
The ‘Oxygen Supply and Delivery Chain’; diagram summarising the process of oxygen supply and delivery, adapted from themes in existing literature (Hill et al. 2009, Sa’avu et al. 2014, Ginsburg et al. 2012, Belle et al. 2010)

Factors contributing to successful oxygen therapy are summarised in the ‘Oxygen Supply and Delivery Chain’ (Fig. 1). A study by Hill and colleagues’ reported on the availability of oxygen in The Gambia [3]. A government-led team visited multiple hospitals and found delivery of oxygen therapy was limited by electricity supplies, oxygen concentrator/ cylinder availability and little use of pulse oximetry. Staffing levels were found to be low and it was reported that no formal training on oxygen administration had been undertaken. Many of these factors were reflected in a study by Sa’avu and colleagues, conducted in rural highland district general hospitals in Papua New Guinea [4]. In this setting, pulse oximetry was only used in the operating theatres, despite oximetry being fundamental to identifying hypoxaemia in all paediatric patients admitted to hospital [5]. Frequent power outages lasting up to eight hours impeded the use of oxygen concentrators. A survey disseminated to mainly DGHs throughout Africa, Asia and South America by Ginsburg and colleagues deduced oxygen cylinders and oxygen concentrators were available ‘most of the time’ in the majority of hospitals [6]. Barriers surrounding the use of pulse oximetry included supply, competition for use and training. The majority of sites had no policies surrounding oxygen administration. Belle and colleagues surveyed oxygen supply and infrastructure in 231 health facilities across 12 African countries [7]. They found of the 231 health centres 99 (42.9%) had an uninterrupted source of oxygen whereas only 55 (23.8%) had a functioning oxygen concentrator. Uninterrupted electricity supplies (necessary for oxygen concentrators) was available in 81 (35.1%) of health facilities.

The east African country of Uganda has a population of 38 million people and is one of the youngest and fastest growing in the world [8]. Understanding oxygen supply and delivery issues in Ugandan healthcare settings for paediatric patients is limited. Healthcare professionals that provide oxygen therapy for paediatric patients are best placed to shed light on oxygen supply and delivery issues.

### Aim

This study aimed to assess on-the-ground barriers to the provision of oxygen therapy for paediatric patients in three government-funded Eastern Ugandan DGHs.

## Methods

### Site selection

The Ugandan healthcare system is divided into care levels, from village health teams to national referral hospitals [9]. This study assessed oxygen supply and delivery in district level hospitals, to understand the state of oxygen therapy affecting large cohorts of paediatric patients. According to the ‘Uganda Hospital and Health Centre 4 Census survey’, they typically serve a population of up to 500,000 and aim to provide comprehensive medical and surgical services [9]. Three public DGHs in the Eastern Region of Uganda were identified; Pallisa, Atutur and Bududa, each providing hospital-based healthcare to their surrounding areas.

### Site visits

A senior healthcare professional at each hospital was contacted via email. Visits were arranged between 16th and 23rd March 2017. Staff at each hospital were introduced to the study by either a formal introductory talk (Pallisa DGH) or talked through the consent form which described the study (Atutur and Bududa DGHs). Eligibile participants included full or part-time paediatric healthcare staff working at the specific study site i.e. medical officer, nurse or other key informant such as biomedical engineer, manager or head of department. All participants were involved in delivery of paediatric care and administration of oxygen, either directly or indirectly and were willing to be interviewed.

#### Data collection tool development

Existing studies [3, 4, 6, 7], WHO tools [10, 11] and international guidelines [12] relating to oxygen supply and delivery provided a content framework for the production of qualitative data collection tools. A written questionnaire spanning domains of interest including oxygen supply, monitoring and delivery alongside patient assessment and consent for oxygen administration was produced. A pre-pilot focus group discussion/ interview crib sheet covering similar domains and relevant probes was produced, where focus on the individuals’ role with regards to oxygen therapy was established. The crib sheet facilitated the scrutinising of the paediatric admission and treatment process relating to oxygen therapy.

### Pilot work

Piloting of data collection tools was undertaken over a two-day period at Pallisa DGH. The questionnaire was tested but its extended length and the closed, ambiguous nature of the questions restricted responses. Questionnaires were therefore deemed unsuitable as a reliable method of data collection and abandoned. A focus group discussion was carried out at Pallisa DGH using the pre-pilot crib sheet. It was discovered nurses and clinical officers frequently worked independently or in pairs. It was therefore felt individual responses would provide more pertinent information so interviews remained the predominant method of data collection hereafter, although responses from the focus group discussion were incorporated into results. A pilot interview was conducted with a nursing officer at Pallisa DGH. This was an extended length interview and helped establish relevant aspects of oxygen supply and delivery in the setting. Following the pilot interview, the crib sheet was consolidated into an interview framework based around five questions:

- *Can you describe your role with regards to oxygen treatment for paediatric patients?*
- *What factors influence the decision to give a child oxygen?*
- *How is oxygen administered?*
- *Do you face problems when giving oxygen?*
- *How could oxygen therapy be improved?*

To facilitate a directed content analysis technique [13], thirty-nine bullet point topics based upon the pilot interview as well as existing studies [3, 4, 6, 7], WHO tools [10, 11] and international guidelines [12] acted as optional domains for follow-up questions. The semi-structured interviews were designed to elucidate thoughts, opinions and experiences using an inductive approach, allowing for interviewee elaboration. Using a semi-structured interview technique permitted a balance between interviewees discussing local concerns and allowed probing of issues previously highlighted in published literature.

### Data collection

Participants were chosen purposively by their role as direct or indirect providers of oxygen therapy. Convenience sampling meant all eligible available staff members during site visits were approached for interview.

Satisfactory triangulation of responses was achieved by undertaking interviews with multiple healthcare professionals of the same specialty from each site. The interview environment was designed to be non-threatening and conducive to honest answers. Rooms selected were on the hospital site and were private and quiet. Snacks and drinks provided were warmly received by all interviewees, enhancing the interviewer-interviewee relationship. Eligible participants were talked through the consent form, prior to consent form signing and the interviews being conducted.

JD (male) was the sole interviewer. All interviews were audio-recorded. Duration of interviews varied from 8 min (m) 32 s (s) to 38 m 25 s, due to differences in discussion topics and data saturation occurring at an earlier stage as the number of interviews undertaken progressed. All interviews were transcribed by JD, including non-audible detail such as extended pauses, feelings of frustration or assertion. Location and job role were the only identifiable aspects of each transcription, so responses could be grouped accordingly. Repeat interviews and member checking of transcripts was not undertaken.

### Analysis

MAXQDA 12 software was used to collate, store and assist with qualitative data coding and analysis. All transcripts were freely read prior to coding, with each passage of text being coded during the second reading. Codes were defined prior to and during data analysis, based on interview discussion topics, implementing directed content analysis through this ‘deductive category application’ process [13]. Coded passages were re-read and key response themes extracted and summarised. The COREQ tool [14] was used as a self-assessment tool to ensure scientific rigour.

## Results

Twenty-nine participants took part in total, with no dropouts or refusals. Numbers of interviewees varied from site-to-site (Pallisa; *n* = 16 (55%), Atutur; *n* = 7 (24%), Bududa; *n* = 6 (21%)). Seventeen of the twenty-nine participants were nursing officers (59%), six were clinical officers (21%), three were medical officers (10%), two were stores staff (7%) and one was an administrator (3%).

### Oxygen supply and availability

#### Oxygen concentrators

Healthcare professionals at Pallisa DGH were happy to use concentrators when power was available. Frequent concentrator breakdowns with prolonged fixing times by hospital maintenance teams was a problem frequently highlighted.

*“The unavailability could be maybe a certain ward has borrowed it because theirs broke down, not functioning because like now, we don’t have ours, it was taken by another ward. And there is no power”.* Nursing Officer, Pallisa

At Atutur DGH, the frustration from all nursing staff focused on poor electricity reliability, the high burden of patients exceeding the numbers of concentrators available and the frequent need to share the concentrator with operating theatres.

#### Electricity

Healthcare professionals at all sites described power as a “big issue”, constantly being “on and off” and thus oxygen concentrators could not function. This lack of “predictability” was reported as being worse during the rainy season, with reports of power outages lasting for days and sometimes for weeks.

#### Oxygen cylinders

At Pallisa DGH where oxygen cylinders were frequently used as an alternative to the oxygen concentrator, recent improvements were reported including access to working flow-meters, increased cylinder supplies and good communication with ‘stores-men’ when re-fills were required.

*“Pallisa makes sure that there is always oxygen. When the cylinders are empty, they are re-filled”* Nursing Officer, Pallisa

*“I prefer the cylinders….rather than an oxygen concentrator, power goes then you have to keep running up and about looking for the other one”* Nursing Officer, Pallisa

This contrasted with healthcare professionals from Atutur and Bududa DGHs who reported lack of access and availability to oxygen cylinders or lack of access to regulators required to attach to cylinders to facilitate their use.

*“You know in Bududa here, we don’t have the oxygen cylinders, we don’t have”* Nursing Officer, Bududa

#### Patient transfers

Transferring patients to other facilities to facilitate oxygen treatment was reported by both Atutur and Bududa DGHs. The hopelessness of the situation was iterated by many of the respondents;

*“And we have to pay the cost to transfer the patient somewhere to get oxygen, it’s so bad.”* Clinical Officer, Atutur

### Oxygen delivery

#### Establishing an oxygen requirement and the use of pulse oximetry

**Fig. 2:**
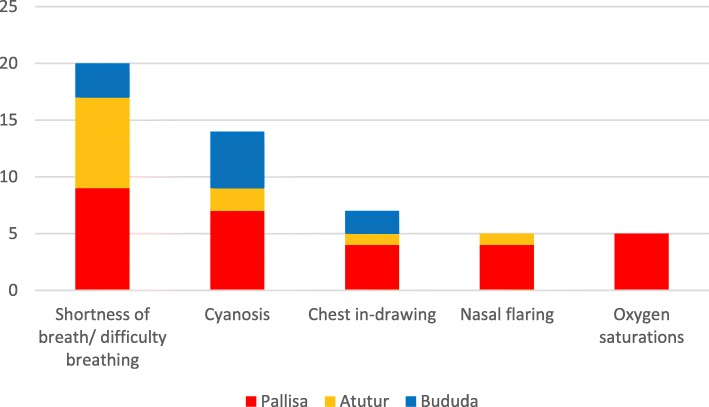
Chart showing number of responses from all three DGHs for symptoms/ signs/ observations used by healthcare professionals to determine an oxygen therapy requirement in paediatric patients

A requirement for oxygen therapy for paediatric patients was established by healthcare professionals at all hospitals using a variety of symptoms, signs and observations (Fig. 2). Where pulse oximeters were not available the three most cited symptoms and signs were “shortness of breath/ difficulty breathing”, “cyanosis” and “chest in-drawing”. At Atutur DGH, healthcare professionals found they had to “estimate” oxygen requirements, using “just clinical judgement” feeling pulse oximetry was only there “to be read in books” and “was left out at [medical or nursing] school as it cannot be done in Uganda”. At Bududa DGH, one nurse reported lack of pulse oximetry “affected her ability to practice”. At Pallisa DGH, oximetry was used in addition to clinical symptoms and signs to identify oxygen requirements for paediatric patients, reportedly used on “all sick children”. Regarding access to oximetry, Pallisa nurses felt they had “good access” and felt “very confident” when using pulse oximeters but a clinical officer reported they “had never seen it” and wanted training on its use.

#### Training

All clinical staff had undergone oxygen administration training during their undergraduate training. Respondents felt this had taken place “a long time ago”, and had a lack of updates since then. Some healthcare professionals at Pallisa and Atutur DGHs had undergone further ‘continuing medical education’ (CME) training, but this had taken place 2–4 years prior and had not been undertaken by all staff members.

#### Equipment

At Pallisa DGH, all respondents regularly used nasal prongs. An established liaison process with stores was present, who would calculate ‘average monthly consumption rates’ per ward to ensure a regular order from national medical stores and sterilisation of nasal prongs between patients meant regular access to nasal prongs. Atutur DGH healthcare professionals described how they would adapt single use nasogastric tubes to deliver oxygen. At Bududa DGH, the sole source of oxygen supply was reportedly only in theatre where there was only one paediatric face mask in use that required sterilising between patients.

#### Decision-making

Nursing officers would frequently state it was the doctor or clinical officer who would make the decision to start oxygen therapy. However, if not available, the majority of nursing officers would feel confident to start oxygen in an emergency situation (if available) and then report this back to the clinical officer.

*“when you are in the ward you should be able to assess that child and see does this child want oxygen then you can even discuss with the clinician and the doctor and you go ahead and give*.” Nursing Officer, Atutur

#### Communication

Communication streams between healthcare professionals appeared satisfactory, where-by nurses felt confident in asking for senior assistance from nursing colleagues and clinical and medical officers, as well as with stores-men to ensure oxygen cylinders were replaced when required.

*“I am on good terms with the stores people. I can easily access the oxygen*” Nursing Officer, Pallisa

#### Staffing

The majority of healthcare professionals felt staffing levels were inadequate, reporting on the “scarcity” of doctors, and the difficulty of “close monitoring” due to the poor nurse: patient ratio.

*“Because for us here doctors are scarce so it depends on, you decide by your own because if you wait for a doctor, things will go worse*” Nursing Officer, Pallisa

“*the number of staffs is quite low and therefore we need many health workers so that the oxygen section can be given the necessary attention because it needs the health worker to be on standby all the time so we can monitor the flow rates*” Clinical Officer, Bududa

#### Parent/carer perceptions of oxygen therapy

Healthcare professionals frequently reported fear of oxygen expressed by parents and carers of children:

*“they feel like the child is dying.. fear or phobia of oxygen here”* Clinical Officer, Bududa

*“The majority of them think that...when you mention the word oxygen, it means the end of the child”* Nursing Officer, Bududa

*“they fear you are going to kill the baby [with oxygen]”* Nursing Officer, Atutur

*“maybe last time they put my brother on oxygen and he passed on so they kind of have such feelings”* Nursing Officer, Atutur

*“sometimes you ask the mother and the mother may refuse and you cannot go forward to give the baby oxygen because the mother has refused”* Nursing Officer, Pallisa

### Improvement strategies

**Fig. 3:**
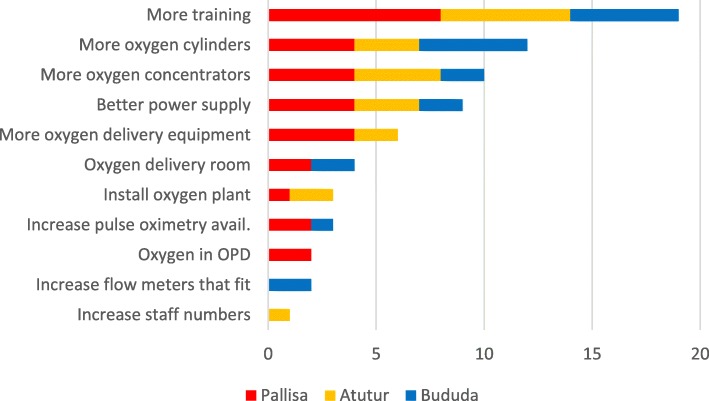
Chart showing number of responses from all three DGHs regarding oxygen supply and delivery improvement proposals (OPD = Out-patient department)

All healthcare professionals outlined what they believed should be done to facilitate improvement of oxygen therapy for paediatric patients in their hospital (Fig. 3). These ideas reflected the barriers to oxygen therapy they had previously discussed.

## Discussion

This study has identified numerous issues relating to oxygen therapy for paediatric patients from the perceptions of healthcare professionals involved in its’ supply and delivery. Across all sites, main barriers highlighted included poor electricity supplies, frequent concentrator malfunctions, low staffing levels and lack of training. At Atutur and Bududa DGHs, these barriers appeared compounded by lack of oxygen cylinders, lack of oxygen concentrators, lack of delivery equipment and no access to pulse oximetry, issues which were reflected in improvement proposals. Parents and carers’ fear of oxygen therapy provided an additional barrier to oxygen delivery to children. Through the prioritisation of oxygen supplies by administrative staff, effective communication between the stores and wards, large back-up supplies of oxygen cylinders with functional flow-meters, a paediatric-specific oxygen concentrator, access to and use of pulse oximetry, healthcare professionals at Pallisa DGH reported use of oxygen therapy that more closely adhered to international recommendations [12].

The oxygen supply and delivery problems highlighted by our study reflect many issues raised in existing literature [3, 4, 6, 7]. There may be a number of reasons for this. In 2014, health spending per capita in Uganda, Papua New Guinea and the Gambia was 48, 107 and 23 USD respectively, contrasting with 4568 USD in the UK [15]. Although direct economy comparison between settings is difficult, prioritising care with these small budgets is challenging.

Oxygen concentrators are frequently advocated as oxygen sources throughout the developing world [16–18], for their relative low cost and ability to ‘make’ oxygen on site. They are the WHO preferred means of oxygen delivery in resource-limited health centres. At all three sites, concentrators were often discussed with frustration due to the frequency of breakdown, time taken for repair work to be undertaken and inadequate power supplies. Enarson and colleagues discuss that although a plausible oxygen supply strategy in their resource-limited setting [17], they advocated the use of flow-splitters with concentrators to address the issue of demand overburdening oxygen supplies [17]. Flow-splitters were not used in any of the hospitals in our study, limiting the number of patients able to receive oxygen from each concentrator. Enarson and colleagues highlight that equipment, training and maintenance must accompany oxygen concentrator systems in order to achieve effective oxygen delivery [17].

The reliance on a stable power supply also presented a large problem for concentrators in all hospitals. A study by Peel and colleagues demonstrated lower voltages and frequent power outages caused electrical faults in concentrators and break down, possibly explaining the problems reported at each hospital in our study [19]. Power supply issues in Uganda were reflected in a World Bank assessment in 2009 [20]. Uganda’s capacity factor- an indicator of power system’s proximity to overload - was calculated at 94%, the highest of all sub-Saharan African countries studied [20]. This problem demands a satisfactory back-up power solution. A study undertaken in a large Eastern Uganda referral hospital concluded solar energy was sufficient to power oxygen concentrator systems to treat children with hypoxaemia and respiratory distress [21] and perhaps could be considered at other hospitals with electricity supply problems.

The role of the nurses was comprehensive with regards to oxygen therapy, corresponding with the contents of the ‘Ugandan National Council for Higher Education Minimum Standards for Courses of Study for the Bachelor of Nursing Science’ [22]. However, this publication highlights use of oxygen funnels, tents and cylinders but does not discuss concentrators. This contrasts with the eastern Ugandan DGHs’ equipment availability and WHO recommendation [23]. National Ugandan Clinical Guidelines [24] also advocate the use of targeted oxygen saturations; however our study indicates this rarely occurs in practice. From an oxygen therapy perspective, national guidelines appear out-of-sync with the reality of Ugandan healthcare available in the DGH setting studied.

Patient/parent perceptions of oxygen in this setting are rarely reported on. Our study has highlighted the fear of oxygen amongst parents of paediatric patients and reflects a study by Stevenson and colleagues [25], who also noted the association between oxygen therapy and death by those interviewed. Education of local populations regarding the benefits of oxygen therapy may improve uptake and reduce fear of oxygen therapy.

Considering the reliance on signs and symptoms at Atutur and Bududa DGHs to establish hypoxaemia, it is concerning that signs and symptoms have been identified as poor hypoxaemia predictors. A study by Rao and colleagues demonstrated that, although poor, chest wall in-drawing, crepitation’s and nasal flaring were the three most sensitive predictors of hypoxaemia and cyanosis and nasal flaring had the best positive predictive value [26]. This partly corresponds with interviewee responses in our study, who cited ‘shortness of breath/ difficulty in breathing’, ‘cyanosis’, ‘chest in-drawing’ and ‘nasal flaring’ as signs indicative of oxygen need. A large prospective study in Kenya involving 15,289 paediatric admissions found 977 (6.4%) had hypoxaemia (SpO2 < 90%) measured by pulse oximetry [27] . In this study, the most predictive signs for hypoxaemia were compensated shock, impaired consciousness, bradycardia (a heart rate < 80 beats/minute) and alterations of normal respiratory pattern including irregular breathing and a respiratory rate > 60 breaths/minute.

Considering the variation in reliability of symptoms used to detect hypoxaemia, emphasis must be placed on routine oximetry use.

### Study limitations

There were a number of limitations to this study. The selected sites were all government-funded district general hospitals in eastern Uganda and therefore external validity of results outside of this setting is questionable.

No formal cost analysis or detailed auditing process of oxygen supply and delivery systems was conducted due to difficulty accessing comprehensive hospital records containing this information.

No comprehensive assessment of power supply was undertaken in this study. However, triangulated insight from local healthcare professionals indicated that they could not rely on their current power supply. This highlights a specific area for quality improvement.

The authors’ involvement with oxygen systems in similar settings to the study prior to carrying out this study may have biased data collection and the interview approach. However, data collection adhered closely to qualitative data acquisition techniques, ensuring reliability and validity [28]. The Delphi technique and member checking would have a been useful techniques to establish consensus responses, strengthening conclusions and enhancing inter-disciplinary view-points [29], but this was not undertaken in our study.

There are greater numbers of coded passages from Pallisa DGH compared to Bududa and Atutur DGHs, biasing responses towards Pallisa DGH. The semi-structured interview design meant some aspects of the supply and delivery chain were not discussed with all interviewees, which may have been mitigated using a structured interview framework. However, this inductive approach allowed response freedom from participants, allowing them to focus on aspects important to them and therefore gave a truer reflection of local issues. These include teamwork, communication, parents’ oxygen fears and patient transfers to other facilities not previously highlighted by other large studies [3, 4, 6, 7].

The coding process in transcript analysis should ideally be undertaken by multiple coders to incorporate multiple opinions [14]. This study was coded by a single individual (JD), which may have increased bias towards expected responses.

Future studies assessing oxygen therapy should strive to understand local problems by interviewing providers of oxygen therapy. In addition, they should undertake a comprehensive site-specific cost analyses of running cylinders and concentrators over specific time periods coupled with reliability tests. This should be alongside assessment of back-up generators, fuel and cylinder transport costs. This will deduce the most suitable oxygen supply for a specific site, which may include cylinders, concentrators, a combination of the two or a local oxygen plant. Linking improvements in oxygen supply to changes in patient outcomes will help target quality improvement efforts, alongside other strategies that aim to reduce paediatric mortality in resource-limited settings [1].

## Conclusions

This study has highlighted numerous barriers to oxygen supply and delivery to paediatric patients in three Eastern Ugandan DGHs, from the points of view of healthcare providers. Healthcare professionals described lack of infrastructure, facilities and training to effectively deliver oxygen therapy but Pallisa DGH reported use of oxygen therapy which closely adhered to international recommendation. Quality improvement work prioritising oxygen therapy at government-funded district general hospital level should focus on site-specific aspects of oxygen supply and delivery and sensitizing communities to the benefits of oxygen. Further research in resource-limited settings surrounding oxygen supply and delivery will establish areas for continued improvement.

## Abbreviations

CME: Continuing Medical Education
DGH: District General Hospital
GAPPD: Global action plan for the prevention and control of pneumonia and diarrhoea
MoH: Ministry of health
MRRH-REC: Mbale Regional Hospital Research Ethics Commitee
SpO2: Peripheral Oxygen Saturations (measured by a pulse oximter)
UCL: University College London
UNICEF: United Nations Children's Fund
WHO: WHO z in Organization
